# Acute Achilles tendon rupture: minimally invasive surgery versus non operative treatment, with immediate full weight bearing. Design of a randomized controlled trial

**DOI:** 10.1186/1471-2474-8-108

**Published:** 2007-11-06

**Authors:** Roderik Metz, Gino MMJ Kerkhoffs, Egbert-Jan MM Verleisdonk, Geert J van der Heijden

**Affiliations:** 1 Department of Surgery, University Medical Centre Utrecht, The Netherlands; 2 Department of Orthopaedic Surgery, Academical Medical Centre Amsterdam, The Netherlands; 3 Julius Centre for Health Sciences and Primary Care, University Medical Centre Utrecht, The Netherlands

## Abstract

**Background:**

We present the design of an open randomized multi-centre study on surgical versus conservative treatment of acute Achilles tendon ruptures. The study is designed to evaluate the effectiveness of conservative treatment in reducing complications when treating acute Achilles tendon rupture.

**Methods/Design:**

At least 72 patients with acute Achilles tendon rupture will be randomized to minimally invasive surgical repair followed by functional rehabilitation using tape bandage or conservative treatment followed by functional rehabilitation with use of a functional bracing system. Both treatment arms use a 7 weeks post-rupture rehabilitation protocol. Four hospitals in the Netherlands will participate. Primary end-point will be reduction in complications other than re-rupture. Secondary end-point will be re-rupturing, time off work, sporting activity post rupture, functional outcome by Leppilahti score and patient satisfaction. Patient follow-up will be 12 month.

**Discussion:**

By making this design study we wish to contribute to more profound research on AT rupture treatment and prevent publication bias for this open-labelled randomized trial.

**Trial registration:**

ISRCTN50141196

## Background

Controversy continues with regard to the optimal treatment for acute subcutaneous Achilles tendon (AT) ruptures. Treatment can be classified into operative (open or minimally invasive/percutaneous) and non-operative. Post operative splintage can be divided into cast immobilisation and functional bracing.

Traditionally open surgical repair of a ruptured Achilles tendon has been the first choice of treatment due to low re-rupture rates and the possibility for functional post-operative splintage [1-4]. But, 34% of patients treated with open repair suffer from complications other than re-rupture, especially wound infection and adhesions [1-5]. In general, the outcome after treatment of a re-rupture is poor, but results following treatment of a deep infection are devastating [6]. Therefore an effort should be made to prevent infectious complications. Many articles on different types of minimally invasive repair techniques (using limited incisions or performed percutaneously) of ruptured AT's have been published [7-14]. But to date, only two 2 randomized trials have been reported [4,15]. In Khan's review on randomized trials complications other than re-rupture were substantially reduced with percutaneous repair techniques but data were very limited. Data on complications using limited incision techniques are even more scant. As minimally invasive techniques differ it is hard to compare other techniques with these numbers. An advantage of most minimally invasive techniques is smaller scars and less damage to the delicate blood supply of the AT. Importantly, in most patients minimally invasive surgery does allow functional rehabilitation [7]. Patients treated by functional rehabilitation after operation rather than cast immobilisation are reported to have a shorter in-patient stay, less time off work and a quicker return to sporting activities. In addition, lower complication rates, including re-ruptures, are reported [1-5].

The main advantage of conservative, i.e. non-operative treatment is elimination of wound complications and intra-operative sural nerve damage. Complications other than re-rupture are reported to reduce to 3% [5]. But, conservative treatment with cast immobilisation has shown to increase the re-rupture rate [1,5] and cast immobilisation induces delayed recovery due to calf muscle weakness as a result of long immobilisation of the ankle joint. In contrast, conservative treatment by functional bracing does allow immediate weight bearing, preventing calf muscle weakness and enabling fast recovery. In three studies conservative treatment of AT rupture with functional bracing did not result in increased re-rupture rates [16-18]. But since only one of these is a randomized trial [17], more high quality data from randomized prospective studies is needed. We hypothesized that compared to surgical treatment, conservative treatment with functional bracing will reduce the absolute risk of complications other than re-rupture with 30%.

## Methods/Design

### Design of study

#### Context

The efficacy of minimally-invasive surgery versus functional conservative treatment of acute subcutaneous Achilles tendon ruptures will be studied in a randomized trial. Four hospitals in the Netherlands will participate in the study, one of them being a university medical centre. The Medical Research Ethics Committee of all the participating hospitals approved the study protocol.

#### Patient selection and informed consent

All patients who report to the emergency department of one of the participating hospitals with an acute Achilles tendon rupture will be considered for entering the study protocol. Inclusion and exclusion criteria are listed in table 1 and will be checked by an emergency room doctor, surgical resident or surgeon. All eligible patients are asked to provide written informed consent.

**Table 1:** Inclusion and exclusion criteria

**Inclusion Criteria**	**Exclusion criteria**
Achilles tendon rupture.	Re-rupture/bilateral rupture/open rupture.
Treatment starts within 72 hours.	Combination with fracture of foot or ankle.
Diagnoses by physical examination: palpable gap and calf muscle squeeze test.	Former application (injection) of local corticosteroids in tendon area.
Age 18–65 years.	Contra-indications for surgery.
Written informed consent.	Physical or mental handicaps that do not allow functional treatment or otherwise interfere with the ability to follow-up on the study protocol.

#### Randomisation and concealment

Randomisation is concealed by a specially designed internet site. Randomisation is in blocks (4 blocks) and stratified by centre. The treatment nature is open labelled for patients, physicians and physiotherapists. During follow-up visits physical examination reveals the allocated treatment to patient and assessor.

#### Interventions

**Figure 1:**
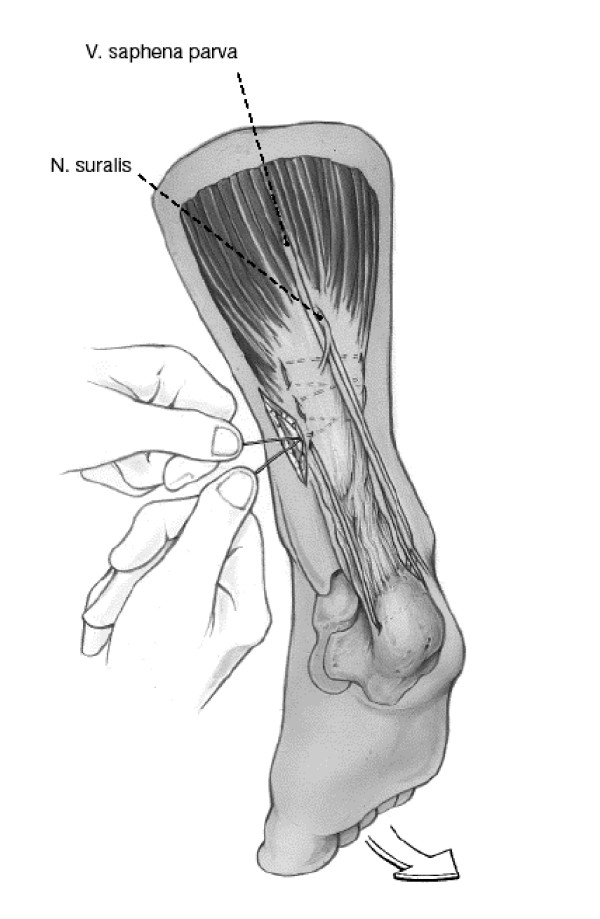
Surgical repair technique. Taken from [7].

Surgical therapy consists of a minimally invasive technique (Figure 1) [7]. The same protocol for the operative procedure was used by all surgeons and residents in the participating hospitals. Also, before study participation all surgeons were familiar with the operative procedure. A less than 5 cm longitudinal incision is made over the posterior aspect of the affected leg just proximal to the rupture site. The incision is slightly medially placed. The subcutaneous fat is divided and the peritendineum opened. Then a Bunell type suture is placed though the proximal end of the Achilles tendon (PDS 1.0). With a hollow mandarin the suture is tunnelled to the lateral aspect of the calcaneal bone and guided out through a 5 mm stab incision. A hole is drilled through the calcaneal bone 1 cm distal to the tendon insertion (exit through 5 mm stab incision medially). The PDS in guided though the hole. Now the mandarin is used to guide the suture back to the proximal site of the tendon. After the foot is placed in plantar flexion the suture is tied. After wound closure a cast is applied with the foot still in plantar flexion. After one week a tape bandage is applied for a total period of 6 weeks. In the first two weeks the tape bandage is supported by a 2 cm heel raise. The following 2 weeks the heel raise is reduced to 1 cm. The last two weeks the heel raise is removed (tape bandage will be renewed every time the heel raise is changed). Full weight bearing is allowed during the 6 weeks of tape bandage, not allowing sporting activities or walking stairs on tiptoes. Crutches are advised in the first week of casting, thereafter for maintenance of balance, but only if necessary.

**Figure 2:**
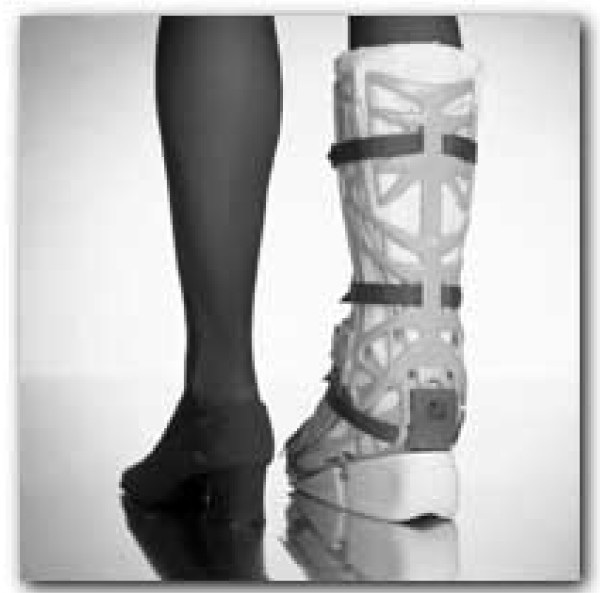
Vacoped.

Conservative therapy consists of a cast in plantar flexion for one week. After one week a functional bracing system (Vacoped, Figure 2) [19] is applied for 6 weeks. The Vacoped bracing system (Company OPED, Valley, Germany) is a multifunctional splint consisting of several components. The essential parts are the dorsal and ventral shell, the vacuum cushion with changeable terry cloth covers, the belts with security locks and the removable sole. Prior to study use the brace was successfully used in a small pilot series and an instructions meeting on brace application was held in all participating hospitals. In the first two weeks the brace is fixed in 30° plantar flexion. The following 2 weeks in is in rigid 15° plantar flexion. The last two weeks the brace is dynamic from neutral position to 30° plantar flexion. Full weight bearing is allowed during the 6 weeks of bracing, not allowing sporting activities or walking stairs on tiptoes. Crutches are advised in the first week of casting, thereafter for maintenance of balance, but only if necessary.

After tape or brace removal patients were advised further rehabilitation with physiotherapist and were allowed sports 3 months after rupture. Patients were free to choose their physiotherapist.

### Design of collection of data

#### Primary endpoint

complications other than re-rupture, i.e. infection, disturbed wound healing, sural nerve injury, scar adhesions, deep vein thrombosis and all other complications per treatment group.

During follow-up all complications will be documented by a surgeon or resident according to a standardised procedure using the definitions of complications presented in the Table 2. All complications will be included in the final analysis of results.

**Table 2:** Definitions of complications used during follow-up.

**Complication**	**Definition**
Infection	Clinical signs of wound infection, i.e. redness, swelling, pain and functional impairment. Deep infection is defined as an infection beyond skin or subcutaneous fat needing surgical treatment in the operating theatre.
Disturbed wound healing	Keloid formation or hypertrophic scar, secondary wound healing, protruding PDS knot.
Sural nerve injury	Any sign of altered sensibility in the sural nerve area diagnosed by surgeon or surgical resident (using touch and pin prick test).
Scar adhesion	Clinical signs of adhesion of skin to underlying tissue layers. Clear wound retraction at ankle movement.
Deep vein thrombosis	Clinical and ultrasonographic signs of deep vein thrombosis of the ipsilateral lower leg.
Other complications	Any complication met during follow-up.

#### Secondary endpoint

re-rupturing (clinical diagnosis supported by ultrasound), time off work, sporting activity post rupture and patient satisfaction. The Thompson test is used for clinical diagnosis of re-rupture. Failure in plantar movement of the foot during calf muscle squeeze is considered a positive sign for re-rupture. Ultrasound evaluation for re-rupture is performed in neutral ankle position. Complete tendon rupture with tendon gap was considered a re-rupture. Time off work will be registered by a patient diary. Complete return to profession was used as endpoint. Stratification to type of profession (sedentary and non-sedentary) will be performed afterwards. A visual analogue scale (VAS) on patient satisfaction with treatment will be measured at 7 weeks, 3 and 12 month. Patient outcome will also be evaluated by the Leppilahti scoring method, a clinical scoring system, including subjective assessment of symptoms and evaluation of ankle range of motion and isokinetic measurement of ankle plantar flexion and dorsifexion strengths (Table 3) [20].

**Table 3:** Leppilahti score.

**Clinical factors**	Scores (points)*
**Pain**	
None	15
Mild, no limitations on recreational activities	10
Moderate, limitations on recreational, but not daily activities	5
Severe, limitations on recreational and daily activities	0
	
**Stiffness**	
None	15
Mild, occasional, no limitations on recreational activities	10
Moderate, limitations on recreational, but not daily activities	5
Severe, limitations on recreational and daily activities	0
	
**Calf muscle weakness (subjective)**	
None	15
Mild, no limitations on recreational activities	10
Moderate, limitations on recreational, but not daily activities	5
Severe, limitations on recreational and daily activities	0
	
**Footwear restrictions**	
None	10
Mild, most shoes tolerated	5
Moderate, unable to tolerate fashionable shoes, modified shoes tolerated	0
	
**Active range of motion (ROM) difference between ankles**	
Normal (<6°)	15
Mild (6°–10°)	10
Moderate (11°–15°)	5
Severe (>15°)	0
	
**Subjective result**	
Very satisfied	15
Satisfied with minor reservations	10
Satisfied with major reservations	5
dissatisfied	0
	
**Isokinetic muscle strength (score)**	
Excellent	15
Good	10
Fair	5
poor	0

* Maximum overall score 100. An overall score of 90–100 rates excellent, 75–85 is good, 60–70 is fair and <55 is poor.

#### Follow-up

Follow-up visits for assessment of primary and secondary endpoints will be scheduled every week during the first 7 weeks. Thereafter, follow-up visits will be planned at 3, 6 and 12 month. Any other consultation for complaints concerning the Achilles tendon area will be documented.

### Design of analysis

Results will be analysed according the intention tot treat principle.

#### Data analysis

The study groups will be compared for their baseline characteristics. The number of complications will be calculated for the primary endpoint. Distribution measures will be calculated for the secondary endpoints at the different moments of follow-up. Differences between groups for the number of complications and distribution of other endpoints will be calculated for each outcome measure with a 95% confidence interval. The study groups will be compared with the chi-square test for categorical outcome variables and the independent sample Student *t *test for continuous outcome variables. The dropouts and withdrawals will be summarized and analyzed by treatment groups. A listing of subject with withdrawal with the date and reasons for termination will be provided.

Because we standardised the intevention procedures we do not anticipate important differences between centers. So we do not stratify our primary analysis for center. However, when eventually differences may occur we will explore their effect in a secondary analysis (Mantel-Haenszel). All analysis in SPSS (SPSS Inc, Chicago Illinois).

#### Sample size

Sample size is calculated on the basis of complication other than re-rupture. With conservative treatment using this new type of functional bracing we hypothesized a 30% reduction in the absolute risk for complications other than re-rupture. This risk reduction is similar to the risk reduction obtained in the systematic review on open versus conservative treatment by Khan: risk of complications for open repair being 34% [5]. Prospective data on the risk of complications of minimally invasive repair is very scant. The Khan review provides the best empirical estimate for the complication risk associated with surgical repair. Therefore we decided to use this risk estimate of complications of open repair for the sample size calculation. With a one-sided α of 0.05, a statistical power of (1-β) of 0.80, and an attrition rate of 10% we need to randomize at least 36 patients per treatment arm.

## Discussion

This study is primarily designed to evaluate the effectiveness of conservative treatment of acute AT ruptures, using a functional bracing system, in reducing complications other than re-rupture. A comparison is made between this functional bracing system and a minimally invasive operative repair of acute AT ruptures. Both treatment options used in this comparison allow immediate full weight bearing so none of the patients is denied the purported advantage of a functional after treatment [2,5,21-24].

There have been randomized clinical trials on treatment of acute Achilles tendon rupture but the methodological rigour is often low. There is a need for more rigorous designed studies on AT rupture treatment as this subject is still very much under debate. By publishing our protocol we wish to show our care for a profound design and methodological quality of our protocol. Moreover, when the design of a study is published it will help to achieve transparency about why and how studies are undertaken. The publication of a study design may help to reduce the problem of publication bias, i.e. selective publication of positive associations and disregarding negative and weak associations, prevent unnecessary duplication of research efforts and duplicate publication [25]. To our knowledge, there has never been a design study published regarding treatment of AT ruptures. By making this design study we wish to contribute to more profound research on AT rupture treatment and prevent publication bias for this open-labelled randomized trial.

## Competing interests

The author(s) declare that they have no competing interests.

## Authors' contributions

RM main author of study design.

GK first initiated the trial and participated in designing the treatment protocol for operative and non-operative treatment.

EV first initiated the trial. Especially involved in all clinical aspects of the trial and study design.

GH contributed to methodology of the study design.

All authors read and approved the final manuscript.

## Pre-publication history

The pre-publication history for this paper can be accessed here:


